# On the State of the Liver in Many Cases of Icterus

**Published:** 1831-04-01

**Authors:** 


LA CHARITE.

### On the State of the Liver in many Cases of Icterus. By M. Corbin.

Icterus is a disease which is very common, but the causes of the yellow suffusion on the skin, are often very obscure. When evident symptoms of gall-stones do not exist, we have nothing but conjecture as to the change in the regular current of the bile. It may be viscid bile plugging up the ducts—or tumours pressing on them—or inflammation of the duodenum — or other causes of which we are ignorant. The disease is not often fatal, and therefore dissection has not thrown so much light on the pathology of the complaint as on that of some others. It has even been discussed in a "concours" at Paris, whether the liver itself be always the seat of the cause which induces icterus. We know, indeed, that jaundice will suddenly arise from strong emotions of the mind; but surely this must be from derangement of the biliary apparatus. But to come to facts.

Out of 1800 patients in the wards of La Charité, between the 1st of January, 1828, and the 1st of April, 1830, 22 were cases of icterus. From this number were excluded all cases of fugitive or slight tinges of yellow on the skin, from temporary bilious affections or indigestion. The cases were all regular icterus. Ten of them were females and twelve males. Fifteen were cured and seven died — a very great proportion in the mortality, as compared with experience in this country. Some of these seven became complicated with other severe affections before death — others presented nothing but the jaundice. The following is a concise exposé of these seven cases.

1. A young man, 22 years of age, died on the 22d August, 1828, in consequence of small-pox, which had come on a short time after the commencement of intense jaundice. The liver was of a yellower colour than usual, and presented brown granulations interspersed in its substance. The biliary ducts were quite free from obstruction.

*Case* 2. An old man of 64, entered the wards with jaundice on the 13th Feb. 1830, and died of peripneumony of the right lung on the 7th of March. The jaundice had nearly disappeared before death. The liver was of a pale yellow colour; but the biliary ducts were free from obstruction.

*Case* 3. A female was affected with encysted ovarian dropsy of both sides for six years. She was seized with icterus ten days before her death, and the complaint had become more and more intense up to that time. The liver had been compressed by the ovarian cyst, and was softer than natural. The organ was pale yellow; but no obstruction could be detected in the ducts.

*Case* 4. An unmarried female, aged 30 years, had been twice in La Charité for liver affection, and ultimately died there on the 1st of May, 1829. During life, the liver could be felt projecting below the ribs, and appeared tuberculated to the touch. There was ascites, and for four months before death decided jaundice—slight at first, but gradually and regularly becoming more intense.

On dissection, the liver was found enlarged, lobulated, interspersed with round bodies of encephaloid substance, and gorged with bile. Yet the biliary conduits were free from obstruction.

*Case* 5. A man, 63 years of age, entered the hospital on the 27th of April 1828, in a state of extreme emaciation and debility. He had cough, and other symptoms of pulmonary affection; but he complained chiefly of pain in the right hypochondrium, which he said, was of long standing. The liver appeared to be considerably enlarged. He was intensely jaundiced.

On dissection, the liver was found to be of great size, and full of tubercles, some of them softened down, and containing a liquid, or soft encephaloid substance. The gall-bladder was enormously distended with green bile. The biliary ducts were entirely free from obstruction.

*Case* 6. A man, 65 years of age, entered the hospital on the 15th November, 1829, with intense jaundice. He was in such a state of prostration, that little information could be elicited from him. It was ascertained, however, that the jaundice was of fifteen days standing. He died three days afterwards.

*Dissection.* There was some yellow serum in the cavity of the abdomen. There was redness, but no ulceration in the mucous membrane of the stomach and bowels. The liver rose very high in the right side of the chest, without any apparent cause. It was larger than natural. It was full of tubercles of various sizes, consistencies, and colours. The gall-bladder was distended with bile—but the biliary ducts were pervious and quite free from obstruction.

*Case* 7. Louis de Moiser, aged 74 years, (formerly Counsellor of Embassy) entered LA CHARITÉ on the 24th Feb. 1828, his whole surface of a beautiful yellow colour — belle couleur jaune. This had been of ten days' duration. He had lost his appetite, was costive, and extremely weak. The urine was green — the liver appeared enlarged. Leeches were applied, and some medicines were prescribed, which appeared to be of use; but on the 9th of March a diarrhœa occurred and continued for some days — then ceased. After this the legs became œdematous — ascites supervened — and on the 28th of the same month he died.

*Dissection.* We shall pass over the minute details of pathological appearances unconnected with the present subject. The duodenum was united with the liver, or rather with a cancerous mass appertaining to the liver. "Le duodenum fait corps avec le foie, ou plutôt avec une masse cancéreuse qui tient au foie." This mass was the size of one's fist. The nature of it is somewhat obscurely described. It included part of the pancreas, the biliary ducts, the cervix of the gall-bladder, and a portion of the concave surface of the liver. The biliary ducts were annihilated—or at all events, totally impervious. The gall-bladder was converted into a solid mass, somewhat resembling the substance of the cancerous tumour above-mentioned. In its centre was found a small quantity of inspissated bile. The liver itself was studded with encephaloid tubercles. From each incision made into its substance, a quantity of aqueous bile issued forth.

*Remarks.* We agree with the author of the report that, in only the last case is there a mechanical obstruction to the flow of bile in its natural course to the duodenum, and consequently an unequivocal cause of the icterus. But we much doubt whether an accurate examination would not have detected mechanical obstructions to the course of the bile in some of the other cases. A canal may be obstructed by the contiguity of a tubercle, without the obliteration of that canal; and in this way we have no doubt, that many instances of jaundice are produced. *Archives.*

